# Thermal Insulation Performance of Silica Aerogel Composites Doped with Hollow Opacifiers: Theoretical Approach

**DOI:** 10.3390/gels8050295

**Published:** 2022-05-10

**Authors:** He Liu, Jia’ao Liu, You Tian, Junhua Jiao, Xuehong Wu

**Affiliations:** School of Energy and Power Engineering, Zhengzhou University of Light Industry, Zhengzhou 450002, China; 332121020779@email.zzuli.edu.cn (J.L.); 332021020682@email.zzuli.edu.cn (Y.T.); 331921010473@zzuli.edu.cn (J.J.); wuxhh@zzuli.edu.cn (X.W.)

**Keywords:** hollow spheres, silica aerogel composite, thermal radiation, modeling, thermal insulation

## Abstract

Silica aerogels demonstrate great promise in thermal insulation applications, such as energy-efficient buildings, cold-chain transportation, and aerospace engineering. However, the application of pure silica aerogels is limited in high temperature applications (>500 K) due to their transparency in the wavelength of 2–8 µm. The conventional strategy is to dope silica aerogel with solid spherical opacifiers (e.g., SiC, TiO_2_, and ZrO_2_) to increase their extinction coefficient; however, incorporating solid opacifiers into silica aerogel matrix improves the structural density of silica aerogel composites. Herein, we propose to improve the extinction coefficient of the silica aerogel by using hollow opacifiers. A theoretical model was developed to investigate the parameters including the outer diameter, shell thickness, and mass fraction on both the radiative thermal conductivity and total thermal conductivity of the silica aerogel composite doped with hollow opacifiers. Our results indicate that doping hollow opacifiers can enable the silica aerogel matrix to achieve lower radiative thermal conductivity when compared to matrices doped with optimally sized solid opacifiers. The total thermal conductivity of silica aerogel doped with hollow opacifiers could be lower than that of the silica aerogel doped with optimally sized solid opacifiers. This work contributes to the understanding of heat transfer within porous materials and guides the structural design of high-temperature thermally insulating materials.

## 1. Introduction

Nanoporous silica aerogels, which have many prominent properties such as low density (3–500 kg/m^3^), large specific surface (500–1000 m^2^/g), and ultralow thermal conductivity (0.010–0.021 W/(m·K), 300 K) [1,2,3], show promising applications in zero-emission buildings, cold-chain transportation, thermal energy storage, and aerospace. In room environments (300 K, 1.0 atm), the thermal conductivity of silica aerogel with a density of ~110 kg/m^3^ is as low as 0.013 W/(m·K) [3], which is only 50% of that of still air. However, pure silica aerogel is transparent to electromagnetic waves in the wavelength range of 2–8 μm [4], leading to a poor thermal insulation performance at high temperatures (>500 K). Generally, spherical opacifiers with high extinction coefficient in the short-wavelength range, such as carbon black, SiC, TiO_2_, ZrO_2_, and Al_2_O_3_, are adopted to reduce the thermal conductivity of pure silica aerogel at high temperatures [5,6,7,8,9,10]. For example, Feng et al. [7] experimentally showed that the extinction coefficients of silica aerogel doped with a 25% mass fraction of SiC particles increased from 1.9–12.6 m^2^/kg to 52.7–58.8 m^2^/kg in the wavelength range of 2–7.5 μm. Paik et al. [11] found that the effective thermal conductivity of aerogel composite doped with TiO_2_ powders with a diameter of 1–2 μm and a concentration of 100 mg/m^3^ could be reduced from 0.140 to 0.026 W/(m·K) at 1073 K. Although increasing the mass fraction of opacifiers reduces the radiative thermal conductivity of aerogel composites, the solid thermal conductivity is also improved due to the high thermal conductivity of the opacifiers. For example, the thermal conductivity of SiC at ambient conditions is 83.6 W/(m·K), which is approximately 6400 times higher than that of silica aerogels. Therefore, the total thermal conductivity decreases and then increases, and there exists an optimized doping amount [12]. Besides, the density of opacifier, for example, SiC (3100 kg/m^3^), is much higher than that of pure silica aerogel [12]. Doping low-density silica aerogels with high-density solid opacifiers increases the density of the aerogel composite, thus limiting its applications where lightweight materials are preferred, such as aerospace, for example.

In recent years, micro/nanoscale hollow spheres have attracted great interest in thermal management due to their unique advantages such as low density, low thermal conductivity, and improved light management [13,14,15,16]. For example, recent work shows that [17] hollow-grained La_2_Zr_2_O_7_ ceramics with an outer diameter of 240 nm and a shell thickness of 20 nm can achieve a low thermal conductivity of 0.0126 W/(m·K) at 1073 K, which is >100 times lower than the amorphous limit of bulk La_2_Zr_2_O_7_. Due to the air cavity inside the hollow spheres and multiple reflections between the outer and inner surfaces, the light reflectivity of hollow TiO_2_ spheres with an external diameter of 1–2 µm and shell thickness of 0.2–1.0 µm is two times larger than that of solid TiO_2_ spheres with the same size in the wavelength range of 200–800 nm [6,18]. Meanwhile, incorporating hollow spheres to the solid matrix can improve the thermal insulation properties and reduce the structural density of the material. For example, adding hollow glass microspheres with a volume fraction of 0.44 to a waterborne polyurethane matrix can reduce 45.2% (50.9%) of the material’s thermal conductivity (density) [19]. These results indicate that hollow spheres can effectively suppress thermal transport via heat conduction, showing promising potential for lightweight super-insulating materials. 

In this work, we developed a theoretical model to investigate the effective thermal conductivity of silica aerogel composite doped by hollow opacifiers, in which factors such as outer diameter, inner diameter, and the mass fraction of the doped hollow opacifiers were considered. This work demonstrates a facile yet effective strategy to design lightweight super-insulating materials for high-temperature thermal insulation applications.

## 2. Results and Discussion

**Figure 1:**
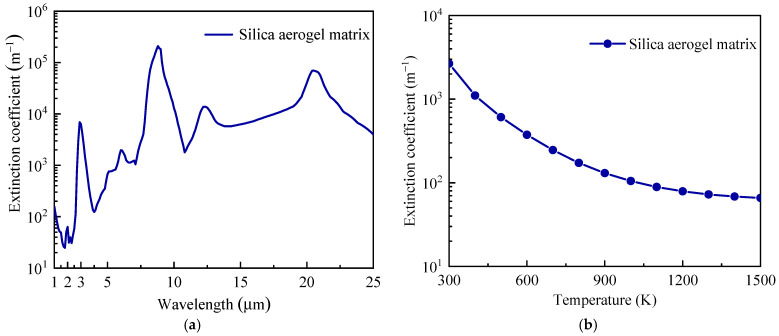
Radiative properties of silica aerogel matrix with a density of 130 kg/m^3^. (**a**) Extinction coefficient vs. wavelength, and (**b**) Rosseland mean extinction coefficient vs. temperature.

In the following, we first validated the developed model for predicting radiative properties of hollow opacifiers. Then, the effect of the geometric parameters, including the size (inner and outer diameters) and mass fraction of hollow opacifiers, on the radiative and total effective thermal conductivity of the silica aerogel composites was discussed. Hollow opacifiers are assumed to be filled with air. According to [8,10], a silica aerogel matrix with a density of 130 kg/m^3^ demonstrated the lowest thermal conductivity, and SiC powders with a high extinction coefficient and high temperature stability were adopted as the opacifiers in this work (Table 1). The refractive index of SiC can be found in [12]. The spectral extinction coefficient of the silica aerogel matrix (Figure 1a) was obtained from [20]. Figure 1b shows the calculated Rosseland mean extinction coefficient of silica aerogel matrix as a function of temperature. It was seen that the Rosseland mean extinction coefficient dramatically decreased as temperature increased.

### 2.1. Validation

**Figure 2:**
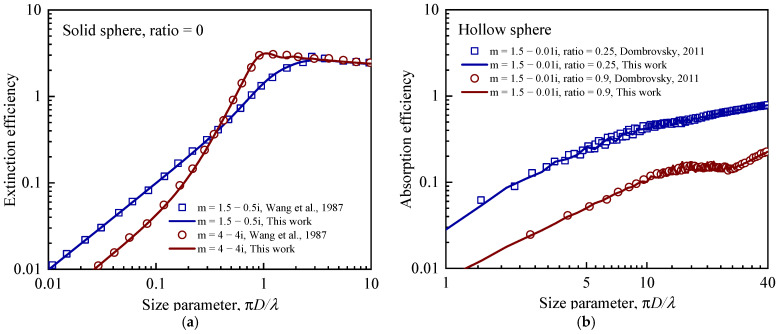
Comparison of radiative properties of solid and hollow spheres with those from the literature [21,22]. (**a**) Extinction efficiency of a solid sphere vs. size parameter, and (**b**) Absorption efficiency of a hollow sphere vs. size parameter. Adapted with permission from Ref. [21], 1983, Elsevier, and Ref. [22], 2011, Begell House, Inc.

Figure 2a compares the calculated extinction efficiency of a solid sphere with the result from [21], where the complex refractive indices were m = 1.5 − 0.5i and m = 4 − 4i, respectively. The real part of the complex refractive indicates phase velocity, and the imaginary part indicates the attenuation amount of light propagating through the materials. Figure 2b compares the calculated absorption efficiency of the hollow sphere with the measured result from [22], where the complex refractive index was m = 1.5 − 0.01i, and the ratios of d to D were 0.25 and 0.9. It was observed that the calculated radiative properties of both solid and hollow spheres agreed well with the results from the literature, indicating that the developed models in the present work are accurate and reliable.

### 2.2. Influence of Outer Diameter and Shell Thickness on the Extinction Efficiency

**Figure 3:**
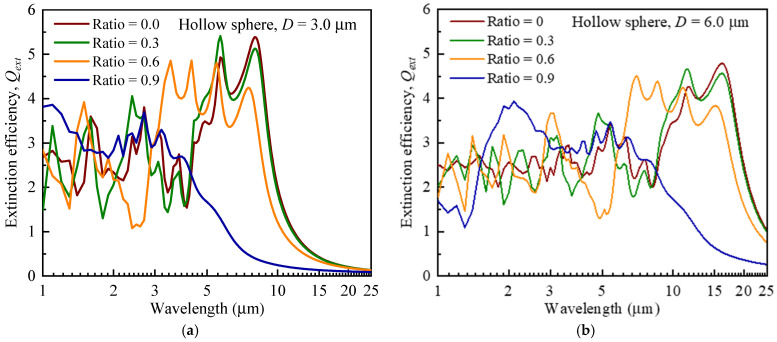

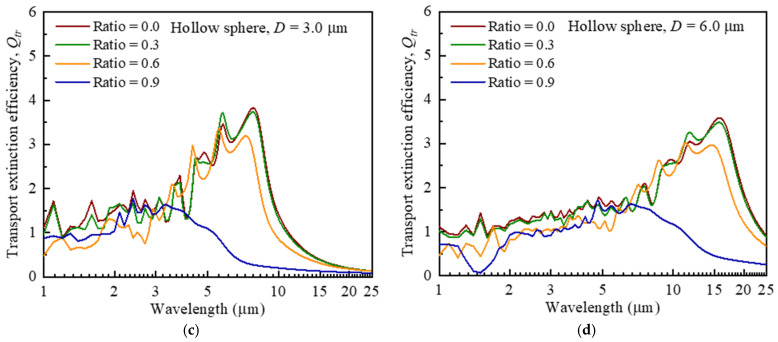
Influence of shell thickness on the extinction efficiency and transport extinction efficiency of hollow sphere. (**a**) Extinction efficiency vs. wavelength, where the outer diameter is 3.0 μm, (**b**) extinction efficiency vs. wavelength, where the outer diameter is 6.0 μm, (**c**) transport extinction efficiency vs. wavelength, where the outer diameter is 3.0 μm, and (**d**) transport extinction efficiency vs. wavelength, where the outer diameter is 6.0 μm.

Figure 3a,b show the variation of the extinction efficiency of a hollow opacifier as a function of wavelength, in which d/D=0.0 (solid), d/D=0.3, d/D=0.6 and d/D=0.9, respectively. The shell thickness equaled (D−d/2.0) and decreased with the increase of d/D. Due to the decrease of the volumetric equivalent refractive index of the hollow opacifier, the peak values of extinction efficiency decreased, and the peak positions moved towards the short-wavelength direction with the decrease of shell thickness. For example, when D=3 μm, the peak positions of extinction efficiency moved from 5–10 μm to 2–4 μm, and the average peak value of extinction efficiency decreases from ~5 to ~3 as d/D increases from 0 to 0.9. A similar trend was also observed when D=6 μm, as shown in Figure 3b. The above trends agree with the results from [23]. Comparing Figure 3a and Figure 3b shows that the peak positions of extinction efficiency move to the long-wavelength direction as the outer diameter of the opacifier increases. We note that the extinction efficiency does not consider anisotropic scattering, which may overestimate the extinction effect of the doped hollow opacifiers. Figure 3c,d show the variation of the transport extinction efficiency ($Q_{\lambda ,p}^{tr}$) of a hollow opacifier that excludes forward scattering as a function of wavelength. Here, the transport efficiency is expressed as:

(1) $$Q_{\lambda ,p}^{tr}=(1-\omega_{\lambda }g_{\lambda })Q_{\lambda ,o}$$

where $\omega_{\lambda }$ denotes the scattering albedo, $g_{\lambda }$ is asymmetry coefficient, and $Q_{\lambda ,o}$ is spectral extinction coefficient of a hollow opacifier.

Comparing Figure 3a,b with Figure 3c,d, the transport extinction efficiency is lower than that extinction efficiency in the wavelength range of 1–25 µm. Similarly, the peak values of transport extinction efficiency decrease and the peak positions of transport extinction efficiency shift toward the short-wavelength direction with the decreases of shell thickness. In addition, we observe that the hollow structure may demonstrate a higher transport extinction efficiency than that of a solid structure in a specific wavelength range. For example, when D=3 μm, the value of $Q_{\lambda ,p}^{tr}$ of a hollow opacifier with d/D=0.3 is larger than that of the solid opacifier (d/D=0) in the wavelength range of 4–6 µm. The above results show that adopting hollow opacifiers can be an effective way to reduce radiative thermal conductivity at high temperatures, and both the particle size and the shell thickness affects the extinction efficiency.

### 2.3. Influence of Outer Diameter and Shell Thickness on the Radiative Thermal Conductivity

**Figure 4:**
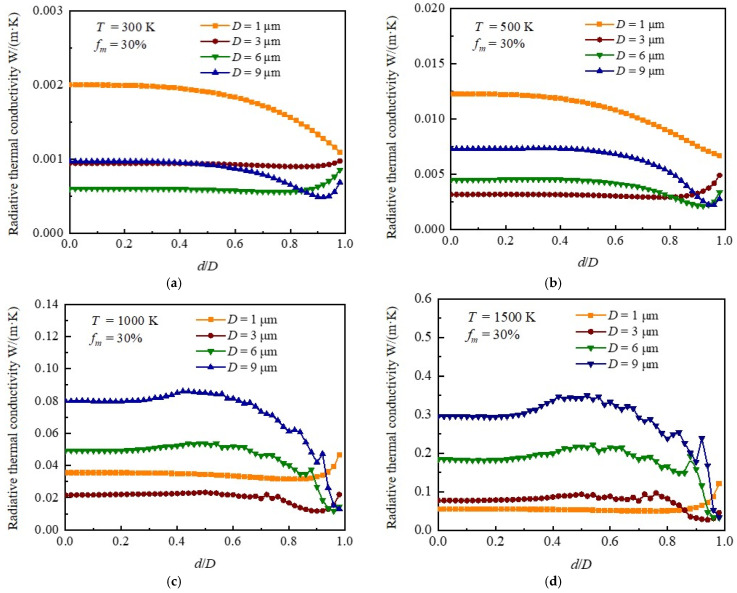
Influence of shell thickness on the radiative thermal conductivity of silica aerogel composites doped with hollow opacifiers with a mass fraction of 30%. (**a**) Radiative thermal conductivity of hollow opacifier-doped silica aerogel at 300 K as a function of shell thickness. (**b**) Radiative thermal conductivity of hollow opacifier-doped silica aerogel composite at 500 K as a function of shell thickness. (**c**) Radiative thermal conductivity of hollow opacifier-doped silica aerogel composite at 1000 K as a function of shell thickness. (**d**) Radiative thermal conductivity of hollow opacifier-doped silica aerogel composite at 1500 K as a function of shell thickness.

Figure 4 depicts the variation of the radiative thermal conductivity of silica aerogel doped with hollow opacifiers with different outer diameters and mass fraction of 30% at various temperatures (300 K, 500 K, 1000 K, and 1500 K) as a function of shell thickness. According to Wien’s displacement law [24], the peak wavelength of thermal radiation shifts towards the short-wavelength direction with increasing temperature. Thus, we observed that the optimal diameter of the solid opacifiers (d/D=0) decreased gradually when the temperature increased from 300 K to 1500 K (Figure 4a–d). For example, as shown in Figure 4a, the silica aerogel doped with 6 µm diameter solid opacifiers demonstrated the lowest radiative thermal conductivity. As the temperature increased to 1500 K (Figure 4d), the lowest radiative thermal conductivity was achieved by the silica aerogel composite consisting of 1 µm diameter solid opacifiers. However, the above trend may not be suitable for hollow opacifiers due to the shift of the peak values of the transport extinction efficiency (Figure 3). The hollow opacifier with a large diameter and small shell thickness can achieve similar or even lower radiative thermal conductivity comparing to small-sized opacifier at high temperature. For example, when d/D≈0.95, the radiative thermal conductivity of silica aerogel doped with hollow opacifiers with an outer diameter of 9 μm was lower than that of silica aerogel doped solid opacifiers with a diameter of 1 µm. The above results indicate that the hollow opacifiers with a large outer diameter and thin shell thickness are also suitable for reducing the radiative thermal conductivity of silica aerogel at high temperature. In addition, we noticed that the variation trends of radiative thermal conductivity of silica aerogel composites doped with hollow opacifiers with respect to shell thickness are closely related to the outer diameter of opacifiers and the working temperature. For example, the radiative thermal conductivity of silica aerogel composites doped with hollow opacifiers with an outer diameter of 1 µm decreased at 300 K and 500 K and increased at 1000 K and 1500 K with a decrease of shell thickness (or increase of d/D). The radiative thermal conductivity of silica aerogel composites doped with hollow opacifiers with an outer diameter of 9 µm decreases then increases with decreasing shell thickness (or increasing d/D) at 300 K and 500 K. Therefore, to achieve minimum radiative thermal conductivity at a specific working temperature, detailed calculations are needed for the selection of hollow opacifiers with an optimal outer diameter and shell thickness. 

**Figure 5:**
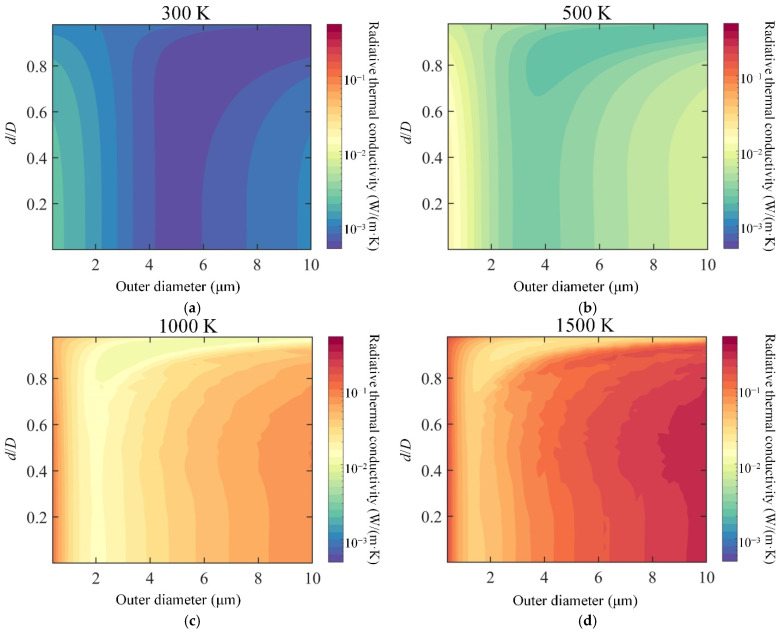
Influence of the shell thickness and outer diameter of hollow opacifiers on the radiative thermal conductivity of silica aerogel composites at a mass fraction of 30%. (**a**) Radiative thermal conductivity of hollow opacifier-doped silica aerogel at 300 K as a function of shell thickness and outer diameter. (**b**) Radiative thermal conductivity of hollow opacifier-doped silica aerogel composite at 500 K as a function of shell thickness and outer diameter. (**c**) Radiative thermal conductivity of hollow opacifier-doped silica aerogel composite at 1000 K as a function of shell thickness and outer diameter. (**d**) Radiative thermal conductivity of hollow opacifier-doped silica aerogel composite at 1500 K as a function of shell thickness and outer diameter.

Figure 5 shows the variation of the radiative thermal conductivity of silica aerogel composite as a function of both shell thickness and outer diameter of hollow opacifiers at different temperatures, in which the mass fraction of the doped hollow opacifier is 30%. As shown in Figure 5a, the optimal diameter of solid opacifiers is 4–6 µm at 300 K. Similar radiative thermal conductivity can be achieved when using hollow opacifiers with an outer diameter of 6–10 um and d/D>0.6. When the temperature increased to 500 K (Figure 5b), the optimal diameter of solid opacifiers was reduced to 3–4.5 µm. More importantly, we see that that silica aerogel doped with hollow structures with an outer diameter of 4–10 µm and d/D>0.7 can achieve lower radiative thermal conductivity. Similar results can also be found in Figure 5c,d, where the temperatures are 1000 K and 1500 K, respectively. In addition, we noticed that the optimal shell thickness of the hollow opacifiers becomes thinner with the increase of temperature, which means that fewer hollow structures can achieve a higher extinction coefficient than solid ones. For example, at 500 K (Figure 5b), the optimal d/D is >0.7, and the optimal d/D needs to be >0.8 when the temperature is 1000 K (Figure 5c). This can be attributed to the shift in the peak positions of transport extinction efficiency observed in Figure 3. Overall, Figure 5a–d indicate that doping of hollow opacifiers allow the silica aerogel to achieve lower radiative thermal conductivity compared to that using solid opacifiers, and the shell thickness reduces as outer diameter increases.

### 2.4. Influence of Mass Fraction on the Total Thermal Conductivity

**Figure 6:**
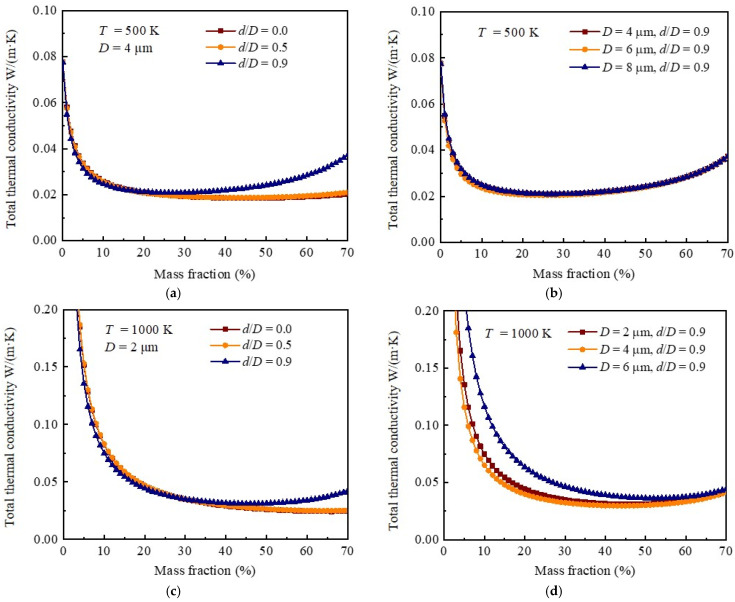
Influence of mass fraction of doped opacifiers on the total thermal conductivity of silica aerogel composite. (**a**) Total thermal conductivity of hollow opacifier-doped aerogel at 500 K as a function of mass fraction, where the outer diameter is 4.0 μm, and *d*/*D* is 0, 0.5, and 0.9, respectively. (**b**) Total thermal conductivity of hollow opacifier-doped aerogel at 500 K as a function of mass fraction, where *d*/*D* = 0.9 and the outer diameters are 4.0, 6.0, and 8.0 μm, respectively. (**c**) Total thermal conductivity of hollow opacifier-doped aerogel at 1000 K as a function of mass fraction, where the outer diameter is 2.0 μm, and *d*/*D* is 0, 0.5, and 0.9, respectively. (**d**) Total thermal conductivity of hollow opacifier-doped aerogel at 1000 K as a function of mass fraction, where *d*/*D* = 0.9 and the outer diameters are 4.0, 6.0, and 8.0 μm, respectively.

Figure 6a depicts the variation of the total thermal conductivity of silica aerogel doped with hollow opacifiers as a function of the mass fraction at 500 K. Due to the competition between the decreased radiative thermal conductivity and increased conductive thermal conductivity, when D=4 μm and d/D=0, we see that the total thermal conductivity decreases as the mass fraction of the doped solid opacifiers increases to around 40%, then increases as the mass fraction continues to increase. A similar trend is also observed when d/D=0.5 and d/D=0.9. In addition, we see that the total thermal conductivity of the silica aerogel doped with hollow opacifiers with an outer diameter of 4 µm and d/D of 0.9 is lower than that of silica aerogel doped with solid opacifiers with the optimal diameter (i.e., 4 µm, Figure 5b) when the mass fraction of the doped opacifiers is <20%. According to Figure 6b, a lower total thermal conductivity can be achieved by doping silica aerogel with hollow opacifiers with an outer diameter of 6 µm and d/D of 0.9 when the mass fraction of the doped opacifiers is <20%. Figure 6c shows the variation of the total thermal conductivity of silica aerogel doped with hollow opacifiers as a function of the mass fraction at 1000 K. Due to the improved radiative thermal conductivity at higher temperature, the optimized mass fraction of doped solid opacifiers at 1000 K is around 60%, higher than at 500 K. We also see that the total thermal conductivity of the silica aerogel doped with hollow opacifiers with an outer diameter of 2 µm (or 4 µm) and a d/D of 0.9 is lower than that of silica aerogel doped with solid opacifiers with the optimal diameter (i.e., 2 µm, Figure 5c) when the mass fraction of the doped opacifiers is <30% (Figure 5c,d). These findings suggest that lower density can be obtained with hollow structures when the target thermal conductivity is the same and better insulation performance properties can be obtained with hollow structures when the mass fraction of doping is the same.

**Figure 7:**
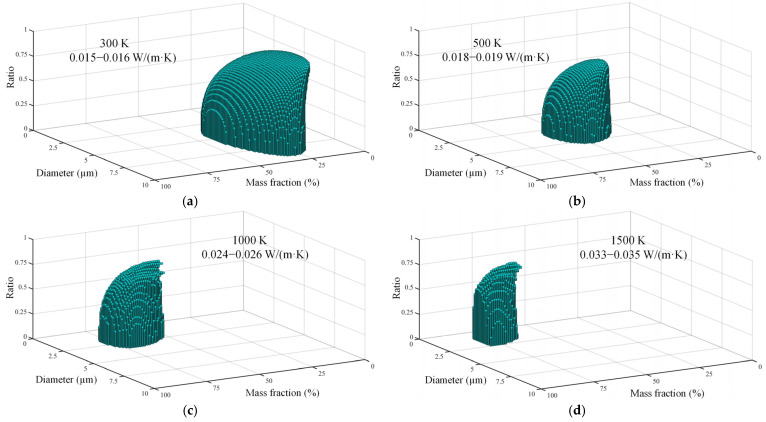
Optimization of geometric parameters of silica aerogel doped with hollow spheres at various temperatures. (**a**) Optimized geometric structures with minimized total thermal conductivity at 300 K. (**b**) Optimized geometric structures with minimized total thermal conductivity at 500 K. (**c**) Optimized geometric structures with minimized total thermal conductivity at 1000 K. (**d**) Optimized geometric structures with minimized total thermal conductivity at 1500 K.

Figure 7a–d depict the optimized outer diameters, shell thicknesses, and mass fractions of hollow spheres doped into silica aerogel at different temperatures. The selected ranges of outer diameter, d/D, and mass fractions were 0.5–10 µm, 0–0.9, and 0–75%, respectively. Similarly to Figure 5, we observe that the optimal outer diameter decreases, and the optimal mass fraction increases with the increase of temperature. More importantly, we confirm that minimal thermal conductivity can be achieved by using hollow opacifiers with lower mass fraction than of solid opacifiers. For example, when the temperature is 1000 K (Figure 7c), adopting hollow opacifiers (2.1 µm, d/D = 0.63) with a mass fraction of 50% can achieve the same thermal insulation performance as using the solid opacifiers with a diameter of 2 µm and mass fraction of 78%. Additionally, it can be seen that that the number of optimal combinations decreases as temperature increases.

## 3. Conclusions

In summary, we developed a theoretical framework to investigate the high-temperature thermal insulation performance of silica aerogel doped with hollow spherical opacifiers, in which factors such as outer diameter, shell thickness, and mass fraction were considered. The calculation of optical properties of a single doped hollow opacifiers was validated through comparison of with the results from the literature. Our modeling results show that as the decrease of shell thickness, the peak positions of the transport extinction efficiency of the doped hollow opacifiers shifts to short-wavelength direction and the average value of the transport extinction efficiency decreases. At a specific temperature, silica aerogel doped with hollow opacifiers can achieve lower radiative thermal conductivity than that of silica aerogel doped with optimally sized solid opacifiers. When the mass fraction of the doped opacifiers is low (e.g., <20%), adopting hollow opacifiers can enable lower total thermal conductivity of silica aerogel composites. This work provides a new strategy to improve the thermal insulation performance of silica aerogels at high temperatures.

## 4. Modeling

**Figure 8:**
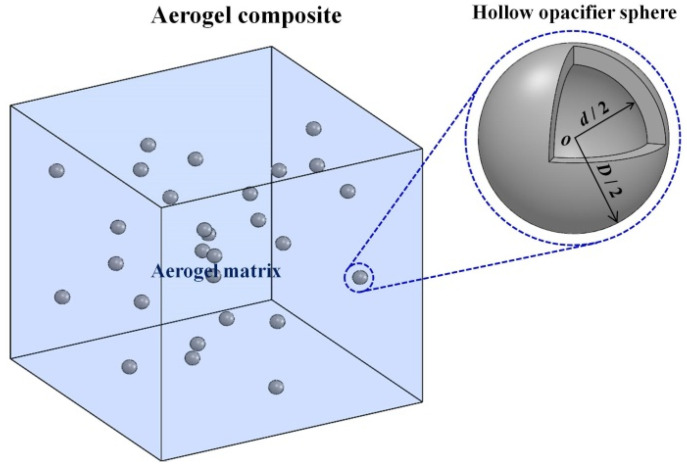
Schematic of silica aerogel composite doped with randomly distributed hollow opacifiers with an outer diameter of D and an inner diameter of d.

Figure 8 depicts the geometric structure of the silica aerogel composite, in which the hollow opacifiers with an inner diameter of d, an outer diameter of D, and a volume fraction of $f_{v}$ were randomly distributed to attenuate thermal radiation. Heat transfer in the silica aerogel composite doped with hollow opacifiers mainly includes heat conduction via solid aerogel matrix, hollow opacifiers, and gas, as well as thermal radiation [3]. The total (effective) thermal conductivity of aerogel composites ($k_{t}$) can be written as,

(2) $$k_{t}=k_{c}+k_{r}$$

where $k_{c}$ is the conductive thermal conductivity and $k_{r}$ is the radiative thermal conductivity [25,26]. 

### 4.1. Conductive Thermal Conductivity of Silica Aerogel Composites Doped with Hollow Opacifiers 

The conductive thermal conductivity of the hollow opacifier-doped aerogel composite can be calculated by the Maxwell model [27]:

(3) $$k_{c}=k_{c,a}\left[\frac{f_{v}^{\frac{2}{3}}+\alpha_{p}\left(1-f_{v}^{\frac{2}{3}}\right)}{f_{v}^{\frac{2}{3}}-f_{v}+\alpha_{p}\left(1+f_{v}-f_{v}^{\frac{2}{3}}\right)}\right]$$

where $k_{c,a}$ is the conductive thermal conductivity of aerogel matrix and $\alpha_{p}=k_{c,o}/k_{c,a}$ is the ratio of thermal conductivity of the hollow opacifiers to that of the aerogel matrix. $k_{c,o}$ can be evaluated by:

(4) $$k_{c,o}=\left(1-{\left(\frac{d}{D}\right)}^{3}\right)k_{s}+{\left(\frac{d}{D}\right)}^{3}k_{g}$$

where $k_{s}$ is the thermal conductivity of the solid shell and $k_{g}$ is the gas thermal conductivity inside the hollow opacifier, which can be calculated by the Kaganer model [15,23,24]:

(5) $$k_{g}=\frac{k_{g}^{o}}{1+2\beta Kn}$$

where $k_{g}^{0}$ is the temperature-dependent thermal conductivity of still air in free space, $\beta =\frac{5\pi }{32}\frac{2-\sigma_{T}}{\sigma_{T}}\frac{9\gamma -1}{\gamma +1}$ is a dimensionless parameter, $\sigma_{T}$ is the thermal accommodation coefficient, and γ is the ratio of the specific heat capacity at constant pressure ($c_{p}$) and the specific heat capacity at constant volume ($c_{v}$). For diatomic gas molecules, γ=1.4 and Kn denotes the Knudsen number, which is the ratio of the mean free path of gas molecules, l, and the inner diameter of the hollow spheres, d.

### 4.2. Radiative Thermal Conductivity of Silica Aerogel Composites Doped with Hollow Opacifiers

The radiative thermal conductivity of hollow opacifier-doped silica aerogel composites is determined by the spectral extinction coefficients of silica aerogel matrix and hollow opacifiers, as well as the ambient temperature. At a specific temperature, the extinction efficiency of a hollow opacifier is determined by the optical properties (i.e., the complex refractive index) and size (e.g., inner diameter and outer diameter) of the opacifier and the wavelength of the incident radiation. To minimize radiative thermal conductivity, the hollow opacifiers with size (i.e., outer diameter) comparable to the wavelength of the incident radiation were selected. Since the volume fraction of the doped opacifiers is relatively low (<10%), each hollow opacifier can be treated as an individual scatterer [28,29,30]. Therefore, Lorenz–Mie theory can be adopted to describe radiative properties of the doped hollow opacifiers (Figure 8), which states [30,31]:

(6) $$Q_{sca,o}=\frac{2}{x^{2}}\sum_{n=1}^{\infty }\left(2n+1\right)\left({\left|a_{n}\right|}^{2}+{\left|b_{n}\right|}^{2}\right)$$

(7) $$Q_{ext,o}=\frac{2}{x^{2}}\sum_{n=1}^{\infty }\left(2n+1\right)Re\left(a_{n}+b_{n}\right)$$

(8) $$Q_{abs,o}=Q_{ext,o}-Q_{sca,o}$$

where $Q_{ext,o}$, $Q_{sca,o}$, and $Q_{abs,o}$ are the extinction efficiency, the scattering efficiency, and the absorption efficiency of a hollow opacifier, respectively, x=kD/2 is the size parameter, D is the outer diameter of the hollow opacifier, k=2π/λ is the the wave number, λ is the wavelength of the incident radiation, Re[·] corresponds to the real part of a complex quantity, and $a_{n}$ and $b_{n}$ are the Mie coefficients which are a function of the inner diameter, outer diameter, incident radiation, and complex refractive index of the solid shell and air inside the voids.

According to the Beer–Lambert law [32,33], the optical transmittance of silica aerogel composite doped with hollow opacifier spheres with a thickness of L can be expressed as:

(9) $$\tau =e^{-\beta_{\lambda }^{tr}L}$$

where $\beta_{\lambda }^{tr}$ is the spectral transport extinction coefficient of aerogel composite, which can be calculated by [8]:

(10) $$\beta_{\lambda }^{tr}=\left(1-f_{v}\right)\beta_{\lambda ,a}+\beta_{\lambda ,p}^{tr}$$

where $\beta_{\lambda ,a}$ is spectral transport extinction coefficient of aerogel matrix and $\beta_{\lambda ,p}^{tr}$ is the spectral transmission extinction coefficient of hollow opacifier, which can be evaluated by considering the anisotropic scattering of hollow opacifiers [34]:

(11) $$\beta_{\lambda ,p}^{tr}=\left(1-\omega_{\lambda }g_{\lambda }\right)\beta_{\lambda ,o}$$

where $\omega_{\lambda }$ denotes the scattering albedo of a single hollow opacifier defined as the ratio of the scattering efficiency to the extinction efficiency, and $g_{\lambda }$ denotes the asymmetry coefficient, which describes the distribution of the scattered radiation in the forward/backward direction and is defined as [30]:

(12) gλ=4y2Qsca,o[∑n=1∞n(n+2)n+1Re(anan+1*+bnbn+1*)+∑n=1∞2n+1n(n+1)Re(anbn*)]

where y=kd/2 is the size parameter, d is the inner diameter of the hollow opacifier, and the asterisk (*) indicates complex conjugation. $\beta_{\lambda ,o}$ is the spectral extinction coefficient of hollow opacifiers doped inside silica aerogel matrix, which is expressed as:

(13) $$\beta_{\lambda ,o}=\frac{1}{4}\pi D^{2}Q_{ext,o}N=\frac{3f_{v}Q_{ext,o}}{2D}$$

where N is the number density of the hollow opacifiers doped in aerogel composite, which can be evaluated by [10]:

(14) $$N=\frac{6\rho_{a}f_{m}}{\pi D^{3}\left[f_{m}\rho_{a}+\left(1-f_{m}\right)\rho_{p}\right]}$$

where $\rho_{a}$ is the aerogel matrix density, $f_{m}$ is the mass fraction of hollow opacifiers, $\rho_{p}=\left[1-{\left(\frac{d}{D}\right)}^{3}\right]\rho_{s}$ is the density of the hollow opacifier sphere, and $\rho_{s}$ is the density of the solid shell. Thus, the volume fraction of hollow opacifiers, $f_{v}$, can be calculated by:

(15) $$f_{v}=\frac{\pi D^{3}}{6}N=\frac{\rho_{a}f_{m}}{\left[f_{m}\rho_{a}+\left(1-f_{m}\right)\rho_{p}\right]}$$

Since radiation energy can only travel over a very short distance within aerogel composites (optically thick medium, optical thickness > 5 [23]), the radiative thermal conductivity can be evaluated by Rosseland approximation [35]:

(16) $$k_{r}=\frac{16}{3\beta \left(T\right)}\sigma T^{3}$$

where T is the ambient temperature and σ is the Stefan–Boltzmann constant. β(T) is the temperature-dependent Rosseland mean extinction coefficient, which is the result of integrating the spectral extinction coefficient over the full wavelength range [8,10],

(17) $$\beta \left(T\right)=\frac{1}{\int_{0}^{\infty }\frac{1}{\beta_{\lambda }^{tr}}\frac{\partial E_{b\lambda }}{\partial E_{b}}d\lambda }$$

where $\lambda_{min}$ = 0.5 μm, $\lambda_{max}$ = 25 μm, $E_{b\lambda }$ is the spectral emissive power of the blackbody, $E_{b}$ is the blackbody emissive power, and the ratio of $E_{b\lambda }$ to $E_{b}$ can be calculated according to [36].

## Figures and Tables

**Table 1:** Materials and properties.

Material	Density (kg/m^3^)	Thermal Conductivity (300 K, 1.0 atm, W/(m·K))
Air	1.29	0.026
Silica aerogel	130	0.013
Silicon carbide (SiC)	3100	83.6

## Data Availability

The data in this work are available upon request from the corresponding author.

## Nomenclature

$a_{n},\;b_{n}$	Mie coefficients
$c_{p}$	specific heat of gas at constant pressure, J/(m^3^·K)
$c_{v}$	specific heat capacity of gas at constant volume, J/(m^3^·K)
D	outer diameter of the hollow opacifiers, m
*d*	inner diameter of the hollow opacifiers, m
$E_{b}$	spectral emissive power of blackbody, W/m^2^
$E_{b\lambda }$	emissive power of blackbody, W/m^2^
$f_{m}$	mass fraction of hollow opacifiers
$f_{v}$	volume fraction of hollow opacifiers
$g_{\lambda }$	asymmetry coefficient
Kn	Knudsen number
k	wave number
$k_{c}$	conductive thermal conductivity, W/(m·K)
$k_{c,a}$	conductive thermal conductivity of aerogel matrix, W/(m·K)
$k_{g}$	gas thermal conductivity inside the hollow opacifier, W/(m·K)
$k_{g}^{0}$	temperature-dependent thermal conductivity of still air in free space, W/(m·K)
$k_{r}$	radiative thermal conductivity, W/(m·K)
$k_{s}$	thermal conductivity of the solid shell, W/(m·K)
$k_{t}$	total thermal conductivity, W/(m·K)
*L*	thickness, m
*l*	mean free path of gas molecules, m
N	number density of the hollow opacifiers
$Q_{abs}$	absorption efficiency of a hollow opacifier
$Q_{ext}$	extinction efficiency of a hollow opacifier
$Q_{sca}$	scattering efficiency of a hollow opacifier
$Q_{\lambda ,p}^{tr}$	the transport extinction efficiency
*T*	ambient temperature, K
*x*, *y*	size parameter
**Greek symbols**	
β	dimensionless parameter
β(T)	temperature-dependent Rosseland mean extinction coefficient, m^−1^
$\beta_{\lambda }^{tr}$	spectral transport extinction coefficient of aerogel composite, m^−1^
$\beta_{\lambda ,a}$	spectral transport extinction coefficient of aerogel matrix, m^−1^
$\beta_{\lambda ,o}$	spectral extinction coefficient of hollow opacifiers, m^−1^
$\beta_{\lambda ,p}^{tr}$	spectral transmission extinction coefficient of hollow opacifier, m^−1^
λ	wavelength of the incident radiation, μm
$\rho_{a}$	density of aerogel matrix, kg/m^3^
$\rho_{p}$	density of the hollow opacifier sphere, kg/m^3^
$\rho_{s}$	density of the hollow opacifier sphere, kg/m^3^
σ	Stefan–Boltzmann constant, 5.67 × 10^−8^ W/(m^2^·K^4^)
$\sigma_{T}$	thermal accommodation coefficient
τ	optical transmittance of silica aerogel composite
$\omega_{\lambda }$	scattering albedo of a single hollow opacifier
